# Program synthesis: challenges and opportunities

**DOI:** 10.1098/rsta.2015.0403

**Published:** 2017-09-04

**Authors:** Cristina David, Daniel Kroening

**Affiliations:** Department of Computer Science, University of Oxford, Oxford, UK

**Keywords:** verification, model checking, synthesis

## Abstract

Program synthesis is the mechanized construction of software, dubbed ‘self-writing code’. Synthesis tools relieve the programmer from thinking about how the problem is to be solved; instead, the programmer only provides a description of what is to be achieved. Given a specification of what the program should do, the synthesizer generates an implementation that provably satisfies this specification. From a logical point of view, a program synthesizer is a solver for second-order existential logic. Owing to the expressiveness of second-order logic, program synthesis has an extremely broad range of applications. We survey some of these applications as well as recent trends in the algorithms that solve the program synthesis problem. In particular, we focus on an approach that has raised the profile of program synthesis and ushered in a generation of new synthesis tools, namely counter-example-guided inductive synthesis (CEGIS). We provide a description of the CEGIS architecture, followed by recent algorithmic improvements. We conjecture that the capacity of program synthesis engines will see further step change, in a manner that is transparent to the applications, which will open up an even broader range of use-cases.

This article is part of the themed issue ‘Verified trustworthy software systems’.

## 1. Introduction

**Figure 1.**
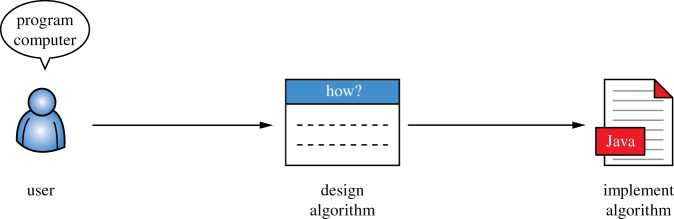
Traditional workflow for using a computer to solve a problem. (Online version in colour.)

A rapidly growing segment of the population has access to computational devices, such as smartphones and computers. Yet, there is a high barrier to entry into programming such devices. The traditional workflow for programming a computer to solve a given problem is illustrated in figure 1 and consists of designing an algorithm that describes how the problem is to be solved followed by implementing this algorithm. Notably, solving a problem with the help of a computer requires expert knowledge of the problem domain as well as of computer programming, which is only available to a very small percentage of all the people who have access to a computer.

**Figure 2.**
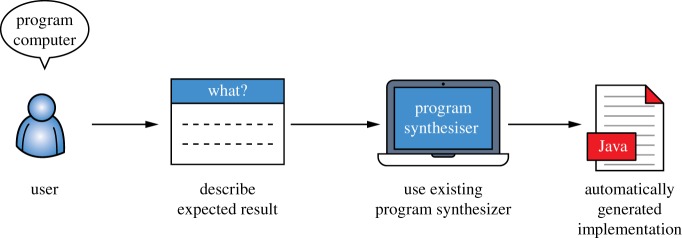
Program synthesis-based workflow for using a computer to solve a problem. (Online version in colour.)

Automated program synthesis has the potential to change the way general-purpose computational devices are used by enabling non-expert users to solve problems in an automated fashion without designing and implementing a new algorithm. Essentially, program synthesis generates an implementation of the program that satisfies a given correctness specification. Consequently, it enables the workflow illustrated in figure 2, where, in order to program a computer to solve a given problem, a user only needs to give a specification of the expected result to the program synthesizer.

Notably, the program synthesis-based approach has the additional advantage that subsequent changes to the problem specification can easily be integrated into the final implementation. Such changes are transparent to the program synthesizer and only require adapting the description of the expected result.

Foundational research in the area of program synthesis has been exceptionally fruitful, beginning with Alonzo Church's work on the Circuit Synthesis Problem in the Sixties [1]. Algorithmic approaches to the problem have frequently been connected to automated reasoning [2,3]. From a logical point of view, a program synthesizer is a solver for second-order existential logic. Second-order existential logic allows quantification over functions as well as ground terms. Consider a formula

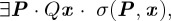

where ***P***^1^ ranges over functions, each *Q* is either ∃ or ∀, ***x*** ranges over ground terms and σ is a quantifier-free formula. A program synthesizer finds a satisfying model for formulae of this kind. Such a satisfying model must map each of the second-order variables ***P*** to some function of the appropriate type and arity. Program synthesis generates programs that compute these functions.

The research community has spent the last decades improving first the techniques for propositional satisfiability (SAT) and later for satisfiability modulo theories (SMT), and as a result, recent methods are able to solve instances of industrial interest. We now have the momentum to advance the two tightly related areas of second-order solving and program synthesis.

*This paper*. In this paper, we will focus on one of the recent directions that greatly raised the profile of program synthesis and ushered in a generation of new synthesis tools, namely counter-example-guided inductive synthesis (CEGIS). We provide an introduction to CEGIS-based techniques and then identify open challenges and discuss some promising directions.

The paper is organized as follows. We start by describing a few applications where program synthesis is valuable. We then introduce the CEGIS architecture as well as some of its most successful applications. After discussing the limitations of the state-of-the-art techniques, we suggest some possible directions for future improvements.

## 2. Users of program synthesis

We give exemplars of applications of program synthesis. Program synthesis has direct applications for various classes of users, from software developers to end-users that are not expert programmers: enabling people with no programming background to develop utility programs, helping regular programmers to automatically improve their code and even teaching.

### (a) Software developers

During the development process, very frequently situations arise where long-term code quality is traded for short-term gain. Under the pressure of deadlines, developers find quick solutions for fixing bugs or adding support for new functionality which result in code that is too complex, bloated by duplication and hard to understand. This leads to what is known as technical debt, a metaphor introduced to indicate ‘not quite right code which we postpone making it right’ [4,5]. In order to repay technical debt, developers perform refactoring. Program refactorings are structured changes to existing software which leave its externally observable behaviour unchanged. They improve non-functional properties of the program code, such as testability, maintainability and extensibility while retaining the semantics of the program. Program synthesis provides a natural way to automate refactoring decisions based on program semantics [6].

Besides code refactoring, software developers can use program synthesis to verify the correctness of their code. Program synthesis has been used to check termination/non-termination of programs, as well as to prove program safety and find bugs [7–10].

### (b) Control engineers

Another use case for program synthesis is represented by the area of embedded systems. A wide range of modern machines rely on *digital controllers*; for example, cars, TVs and even toasters contain fully functional CPUs and are controlled by software. Implementing a digital controller for such a system is a difficult task as it requires understanding the subtle interaction between the continuous behaviour of the physical machine and the discrete behaviour of the controller. Program synthesis can be used by control engineers to automatically produce correct implementations of digital controllers. Examples of applying synthesis techniques to such controllers are [11], where the authors synthesize correct-by-design embedded control software in a Matlab toolbox, and [12,13], where Abate *et al.* focus on the synthesis of digital feedback controllers.

### (c) Non-expert programmers

Millions of people worldwide use spreadsheets for storing and manipulating data. Most of these users are not expert programmers and they often need to create small scripts to perform business tasks. The difficulty is that spreadsheet systems, like Microsoft Excel, come with a multitude of features, and users struggle to find the correct way to accomplish their tasks. More significantly, programming is still required to perform some of these data manipulation tasks. The popular Flash Fill feature released in Excel 2013 is based on program synthesis and helps users to manipulate data via examples [14].

Another application of program synthesis targeting end-users is a method for automatically synthesizing ruler/compass based geometry constructions starting from declarative specifications [15]. The goal is to construct a set of geometric objects *O* with desired properties *φ*_2_ from an initial set of objects *I* with certain properties *φ*_1_. The construction is done using a series of steps *S*, where each step involves using ruler or compass to create new objects. The connection to program synthesis is that objects *I* and *O* can be seen as program variables and *S* is the program whose instruction set includes ruler/compass operations. Synthesizing the geometric construction *S*, given precondition *φ*_1_ and postcondition *φ*_2_, is the program synthesis problem.

Yet another venue for synthesis-based techniques is education, where it is used for the generation of tutorial systems capable of automatically producing problems, solutions and feedback [16]. Generating fresh problems that have specific solution characteristics (e.g. difficulty level, use of a certain set of concepts) is a tedious task for the teacher. Automating it can help prevent plagiarism as each student can be provided with a different problem with the same characteristics. Also, automating feedback generation can save teachers time, as well as enabling consistency in grading.

## 3. Counter-example-guided inductive synthesis

A traditional view of program synthesis is that of *deductive* synthesis from complete specifications [17]. A complete specification eliminates the need for further refinement of the synthesized program, but such specifications are often unavailable, difficult to write, or computationally expensive to verify using automated verification techniques. This has led to the proposal of *inductive* synthesis, which uses as starting point incomplete descriptions of a problem such as test cases, desirable and undesirable behaviours of a software, input/output examples, computation traces of a program for particular inputs and so forth [18]. Inductive methods produce a generalization of such an incomplete specification by identifying general patterns in the data. While inductive synthesis has the flexibility of incomplete specifications, the result may fall short of expectations. In particular, while the final solution reported by such a technique is guaranteed to be correct for the incomplete specification that was provided, it might not exhibit the behaviour expected by the user for cases that are not covered by this specification.

In order to restore soundness, a more recent, counter-example-guided, approach to inductive synthesis borrows ideas from counter-example-guided abstraction refinement (CEGAR) [19] and superoptimization [20,21]. This approach uses an iterative process where each iteration performs *inductive generalization* based on *counter-examples* provided by a verification oracle. Essentially, the inductive generalization uses information about a limited number of inputs to make claims in the form of candidate solutions about all the possible inputs. In the absence of complete specifications, oracles are being queried to distinguish between non-equivalent programs [22,23]. This type of synthesis is known as CEGIS.

The connection between synthesis and theorem proving was established in early work on the subject, which, notably, also found that the capabilities of theorem provers were a bottleneck for synthesis [17]. Nowadays, SAT and SMT solvers allow solving industrial-sized instances. Taking lessons from automated software verification, CEGIS techniques make use of advances in theorem proving in order to remove this bottleneck, in particular, by using SAT and SMT solvers as verification oracles.

**Figure 3.**
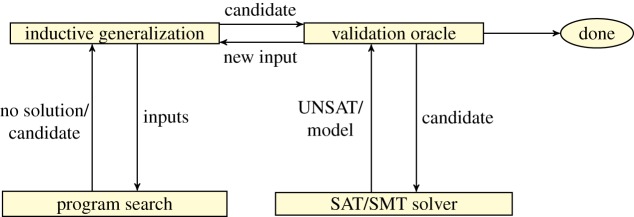
The CEGIS loop. (Online version in colour.)

*Counter-example-guided inductive synthesis architecture*. The general architecture of CEGIS-based synthesizers is given in figure 3 and consists of two phases, the inductive generalization and the verification oracle, which interact via a set of test vectors INPUTS that is updated incrementally. Given some specification, the inductive generalization phase tries to synthesize a candidate solution that works correctly for the current finite set of inputs. Let us recall from §1 that the input specification given to a program synthesizer is a formula of the form

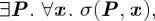

where ***P*** ranges over functions, each *Q* is either ∃ or ∀, ***x*** ranges over ground terms and *σ* is a quantifier-free formula. Note that for simplicity we assume that all the first-order entities are universally quantified. The explanation for this is that formulae with arbitrary first-order quantification can be transformed into this form by existentially quantifying over Skolem functions [24]. Then, the inductive generalization phase searches for a satisfying model for the formula: 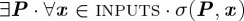. If ***P*** is found, then it is passed to the validation oracle, which attempts to find an input distinguishing the candidate solution from a full solution that works for all possible inputs. If a distinguishing input is found, then it is added into the set of inputs for subsequent iterations. Otherwise, the procedure terminates. The procedure essentially works by iteratively synthesizing new programs that work correctly on more and more inputs.

When synthesizing programs over finite domains, this technique is guaranteed to terminate: it either returns the correct program, or it notes that the components provided are insufficient for synthesizing the correct program. Note that the definition of a program depends on the corresponding problem domain. For illustration, as described in §2, a ‘program’ could be a series of steps for constructing geometric objects, a sequence of instructions for extracting data from spreadsheets, or a plain C/Java program.

An important aspect of any CEGIS-based synthesis algorithm is the manner in which the space of candidate programs is being searched. Next, we describe the most popular strategies, which are run in parallel in [9].

### (a) Explicit proof search

The simplest strategy for finding candidates is to just exhaustively enumerate them all, starting with the shortest and progressively increasing the number of instructions.

### (b) Symbolic bounded model checking

**Figure 4.**
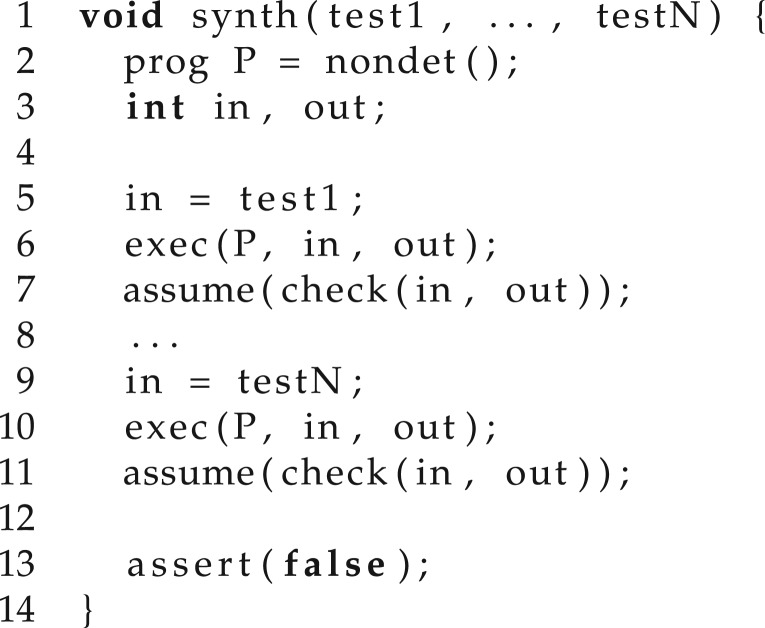
A program that is unsafe if there exists a candidate solution satisfying the specification for all inputs.

Another method for generating candidates is to use bounded model checking (BMC) [25]. Essentially, this strategy requires using the logical specification to build a program that is unsafe (i.e. has an assertion violation) if there exists a candidate solution. For illustration, such a program is given in figure 4 and takes as parameters the test inputs: inputs = {test1, … , test*N*}. Here, nondet provides a nondeterministic program (according to the problem domain), exec is an interpreter for the synthesized programs, check is used to verify that the required specification holds for the current input and corresponding output and assume provides a way of adding assumptions to the program state. The signature of the interpreter is void exec(prog P, int in, int out), where *P* is the synthesized program, in are its inputs and out its outputs. For this example, we assume that the inputs and outputs are integers.

The program contains an assertion which fails if and only if *P* meets the specification for each of the inputs. Finding a new candidate program is then equivalent to checking the safety of such a program. This strategy is complete when synthesizing finite state programs, whose safety is decidable with BMC.

### (c) Genetic programming and incremental evolution

The final strategy is genetic programming (GP) [26,27]. GP provides an adaptive way of searching through the space of programs for an individual that is ‘fit’ in some sense. Commonly, the fitness of an individual is measured by counting the number of tests in inputs for which it satisfies the specification.

To bootstrap GP in the first iteration of the CEGIS loop, a population of random programs needs to be generated. Then, this population is iteratively evolved by applying the genetic operators crossover and mutate. crossover combines selected existing programs into new programs, whereas mutate randomly changes parts of a single program. Fitter programs are more likely to be selected.

Rather than generating a random population at the beginning of each subsequent iteration of the CEGIS loop, GP may start with the population available at the end of the previous iteration. The intuition here is that this population contained many individuals that performed well on the *k* inputs available before, so they will probably continue to perform well on the *k* + 1 inputs available now. In the parlance of evolutionary programming, this is known as *incremental evolution* [28].

## 4. Successful applications of counter-example-guided inductive synthesis

### (a) Program sketching

A successful CEGIS application is program sketching [29,30], where the programmer uses a partial program, called a *sketch*, to describe the desired implementation strategy, and leaves the low-level details of the implementation to an automated synthesis procedure.

The key idea of a program sketch is that difficult expressions and statements are left unspecified. In their place, the programmer describes a space of possible code fragments which the synthesizer can use to complete the missing code. The hypothesis behind sketching is that programmers often have an intuition about the general form of a solution, and this intuition can be used to reduce the space of possible solutions to be considered by the synthesizer.

An example from Solar-Lezama [31] is given below, where each ‘??’ denotes an unknown. The example is attempting to isolate the rightmost 0-bit in a word x. For example, for the word 01010011, the desired result is 00000100, which contains a 1 in the position of the rightmost 0. In order to program this, all one needs to remember is that the solution involved the addition of a constant to x, a negation and a bitwise and.


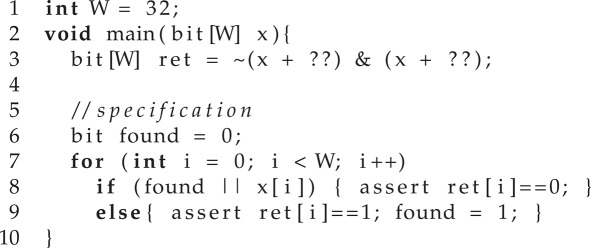


The main procedure is the entry point of the sketch. Then, the synthesizer needs to derive two integer constants *A* and *B* such that, when the first appearance of ‘??’ is replaced by *A* and the second by *B*, the resulting program will satisfy the assertions for all inputs in the input space of main. A valid solution consists of replacing the first appearance of ‘??’ by 0 and the second by 1 in the synthesized code.

### (b) Syntax-guided program synthesis

Following the trend started by sketching, syntax-guided program synthesis (SyGuS) [23] requires the user to supplement the logical specification provided to the program synthesizer with a syntactic template that constrains the space of allowed implementations. The correctness specification of the function *f* to be synthesized is given as a logical formula *φ* that uses symbols from a background theory *T*.

For illustration, consider the problem of synthesizing a program which returns the maximum of two integer inputs. This example is taken from Alur *et al.* [23]. The specification of the desired program max is given by

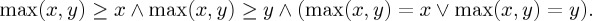


The search space must be suitably defined by an expression grammar which includes addition, subtraction, comparison, conditional operators and the integer constants 0 and 1. Note that, although the integer constants are not required for this particular example, they are provided to the synthesizer in the general scenario where the user does not know the exact form of the solution.

### (c) General-purpose program synthesizers

Many problems can be reduced to program synthesis and then solved with a program synthesizer. In this section, we discuss general-purpose synthesizers that can be used as backends by other tools. While humans do generally have an intuition about the form of a program to be synthesized, we cannot expect the same from tools. Thus, general-purpose synthesizers that are expected to be used by other tools must have a universal target language such that no syntactic restriction of the output needs to be provided.

For illustration, we will look at program analysis problems and how to solve them with the help of a general-purpose synthesizer. Fundamentally, every static program analysis is searching for a program proof. For safety analysers, this proof takes the form of a program invariant [32]; for bug finders, it is a counter-model [33]; for termination analysis, it can be a ranking function [34], whereas for non-termination it is a recurrence set [35]. Finding each of these proofs was subject to extensive research resulting in a multitude of techniques.

An alternative to designing specialized techniques is using a general-purpose program synthesizer to generate programs that compute the corresponding program proof. For brevity, let us consider the case of a program consisting of a single loop with no nesting. We represent the loop by *L*(*G*,*T*), where *G* is the guard and *T* the body such that states *x* satisfying the loop's guard are given by the predicate *G*(*x*). The body of the loop is encoded as the transition relation *T*(*x*, *x*′), meaning that state *x*′ is reachable from state *x* via a single iteration of the loop body. For example, the following loop:


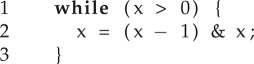


is encoded as:

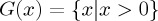


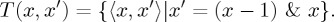


It is customary to abbreviate the sets above using a characteristic function:

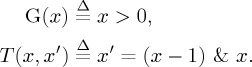


**Figure 5.**
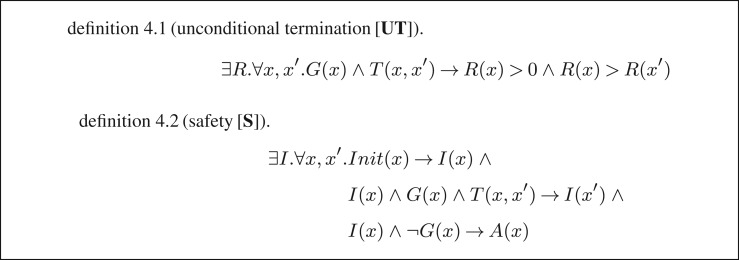
Formulae encoding the termination and safety of a single loop.

To prove (unconditional) termination of *L*, it suffices to find a ranking function for *T ∩ *(*G* *×* *X*), where *X* denotes *L*'s state space, i.e. *T* restricted to states satisfying the loop's guard. The existence of a ranking function is equivalent to the satisfiability of the formula [**UT**] in figure 5: a satisfiability witness is a ranking function and thus a proof of *L*'s (unconditional) termination.

If we use formula [**UT**] as the input specification (where *G* and *T* are replaced correspondingly), then the general-purpose program synthesizer in [9] finds the solution *R*(*x*) *=* *x* for the aforementioned loop.

Now, let us assume we are interested in proving that *L* is safe, i.e. no assertion violation occurs during its execution. We will consider the specification for the same loop *L*(*G,T*) with the addition that *Init* describes its initial states and *A* denotes the assertion immediately after *L*. The corresponding safety specification is given in definition 4.2, where *I* is the safety invariant. This invariant must hold in the initial states, must be inductive with respect to the transition relation *T* and, on exit from the loop, must imply the assertion *A*. Then, proving safety of loop *L* is equivalent to proving the satisfiability of the formula [**S**]. If, as shown below, we augment the previous example with an initial state and an assertion, then the program synthesizer in [9] finds *I*(*x*) *=* *x* ≥ 0 as a safety invariant:


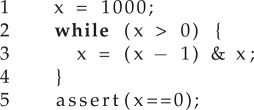


**Figure 6.**
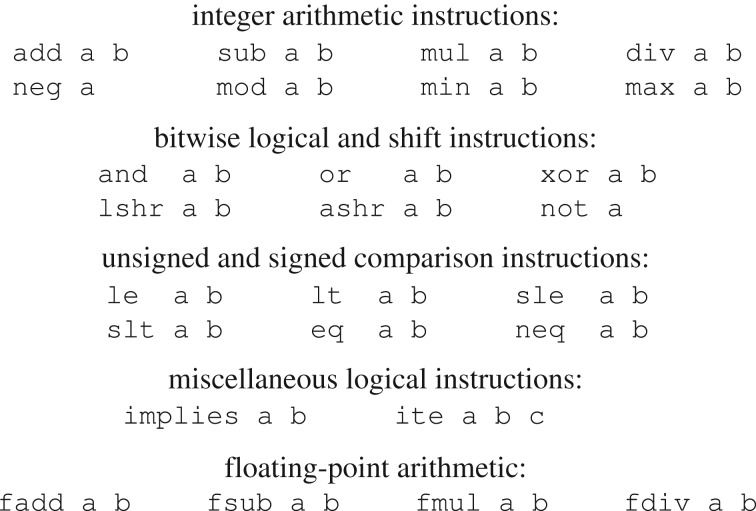
The language 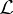 (taken from [9]).

Notably, switching between proving program termination and safety only affects the input specification and it is otherwise transparent to the program synthesizer. Regardless of the specification, candidate programs are written in a simple RISC-like language 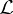, whose syntax is given in figure 6. Operands are either program constants, program arguments or the result of a previous instruction. A program is well formed if no operand refers to the result of an instruction that has not been computed yet. Note that any program in 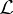 is finite (there is no looping statement) and, additionally, any finite function can be computed by an 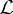-program.

Besides proving program safety and termination, the general-purpose synthesizer in [9] has been used to build tools for checking non-termination of programs [7], finding bugs [8], program refactoring [6] and for synthesizing digital controllers [12,13].

## 5. Challenges of counter-example-guided inductive synthesis-based techniques

While program synthesis has the advantage of generality by providing a unified solution to a very large class of problems, the downside is that it may be challenging for program synthesis-based tools to match the performance of their special-purpose counterparts. While special-purpose tools provide highly specialized search strategy tailored to specific problems, program synthesis-based tools must be generic in their search strategy in order to retain generality.

Currently, the performance issue is being tackled by simplifying the original synthesis problem in order to reduce the space of possible candidates: both sketching and SyGuS require the user to provide some sort of a syntactic template for the desired program in addition to the correctness specification, whereas the universal target language approach only allows synthesizing finite state programs. Thus, while CEGIS-based approaches do represent a significant progress for program synthesis, they are still not ready to be applied to unrestricted real-world problems.

Next, we discuss some of the aspects that, in our experience with CEGIS techniques, are critical for their performance. As mentioned in §3, the general architecture of CEGIS-based synthesizers consists of two phases, the inductive generalization and the verification oracle. We will start with aspects related to the size and exploration of the solution space and then look at the solving process that takes places in both the inductive generalization and the verification oracle. Our conjecture is that further investigation of these aspects will help reduce the performance penalty specific to program synthesis in a manner that is transparent to the user and does not restrict the class of its applications.

### (a) Solution space

#### (i) Avoid unsatisfiable specifications

CEGIS-based synthesizers are generally more efficient at finding satisfying assignments to satisfiable formulae than reporting that a problem has no solution. If the specification formula is unsatisfiable, then the procedure is very likely to not terminate in practice as it must explore all the solution space before coming up with a verdict. Then, writing specifications in a manner that ensures that they are satisfiable has the potential to reduce the solution space that actually gets explored.

For illustration, we will look again at program termination. Recall from §4c that we reduce the termination of a simple loop with no nesting to the satisfiability of the formula [**UT**] in figure 5. The problem that we encountered with this approach was that, in practice, our program synthesis-based termination prover did not come back with an answer whenever *L* was non-terminating.

**Figure 7.**
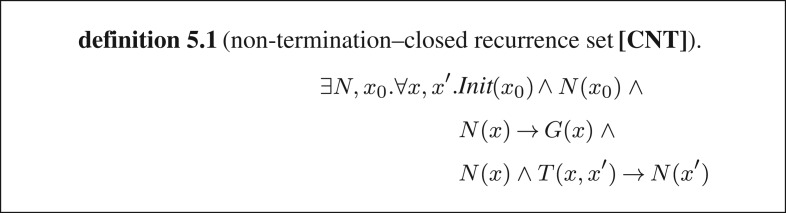
Formulae encoding the non-termination of a single loop.

In order to improve the synthesizer's chances to find an answer, in [7], we augmented the specification with the condition for non-termination. For a deterministic loop, one can prove non-termination by using the notion of a *closed recurrence set* introduced by Chen *et al.* [36]: for each state in the recurrence set *N*, *all* of its successors must be in *N*. The existence of a closed recurrence set is equivalent to the satisfiability of formula [**CNT**] in definition 5.1: *N* is reachable from an initial state *x*_0_, implies the guard and all its successors are still in the recurrence set (figure 7).

Now, we can construct two formulae: one that is satisfiable if and only if loop *L* is terminating and another that is satisfiable if and only if *L* is non-terminating. We will call these formulae *φ* and *ψ*, respectively, and we denote by *N* and *R* the proofs of non-termination and termination, that is recurrence set and ranking function, respectively: ∃*R·∀x*, *x′·φ*(*R*, *x*, *x′*) and ∃*N*, *x*_0_*·*∀*x*, *x′·ψ* (*N*, *x*_0_, *x*, *x*′).

We can combine these: (∃*R·*∀*x*, *x′·φ*(*R*, *x*, *x′*))∨(∃*N*,*x*_0_*·*∀*x*, *x′·ψ*(*N*, *x*_0_, *x*, *x*′))*.*

Which simplifies to: ∃*R*, *N*, *x*_0_*·*∀*x*, *x′*, *y*, *y′·φ*(*R, x, x′*)∨*ψ*(*N*, *x*_0_, *y*, *y*′).

Since *L* either terminates or does not terminate, this formula must have a satisfying model. Thus, either *R* or *N* must exist.

We have used the same approach to build a safety prover/bug finder: a program is either correct as witnessed by a safety invariant or it has a bug shown by an error trace [8]. In both scenarios (termination/non-termination and safety/bug finding), we found that considering only satisfiable instances considerably reduced the percentage of timeouts.

#### (ii) Dynamic instruction set

As mentioned in §4, current CEGIS-based techniques constrain the instruction set and correspondingly the solution space to be searched by supplementing the logical specification with a syntactic template that limits the space of allowed implementations. However, there are two disadvantages to this approach: (i) the applicability of such synthesis techniques is limited to areas where the user knows in advance how the expected solution looks like and (ii) the instruction set is fixed for the whole problem, although certain sub-problems might require different instruction sets.

**Figure 8.**
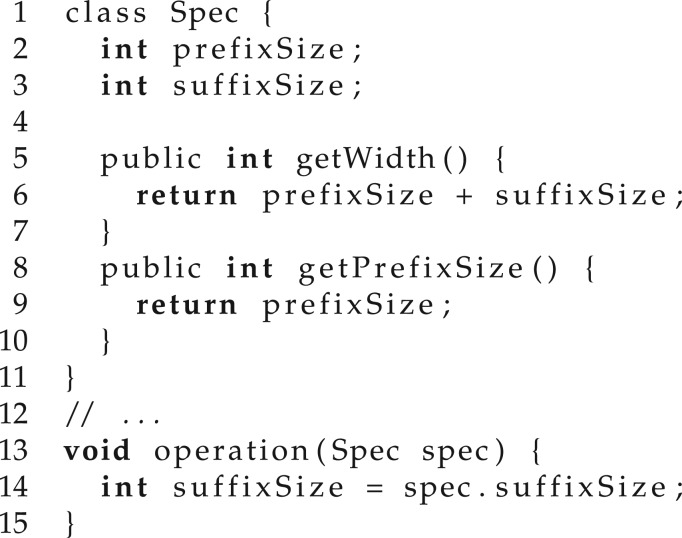
Unnecessary dependency initial.

**Figure 9.**
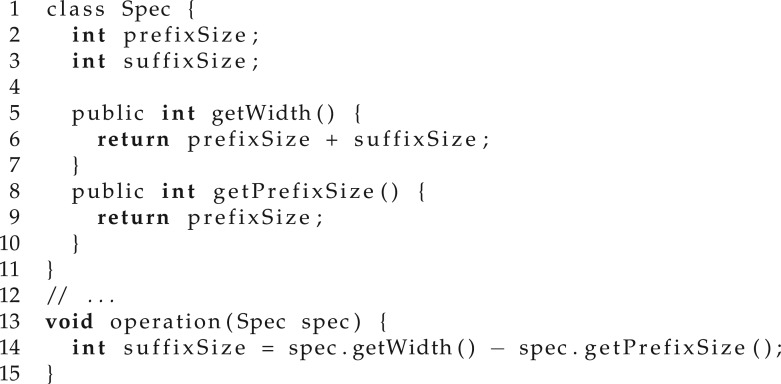
Unnecessary dependency refactored.

For illustration, let us consider that we are using program synthesis for program refactoring (details of program refactoring can be found in §2) and, in particular, let us look at the program in figure 8. Note that, at line 14, method operation depends on member variable suffixSize of class Spec. This is known as an *unnecessary dependency* and can be removed without affecting any of the functionality through the refactoring given in figure 9, where operation depends only on the public methods of class Spec.

When using program synthesis to automatically generate this refactoring, we would want the considered instruction set to be constrained to instructions that return an integer and do not directly access any member variables of class Spec. Note that this will also impact the methods that the synthesizer can call, which, for large applications has the potential to seriously reduce the size of the solution space.

However, we do not want the user to provide any specialized templates/instruction sets. Given that the same tool will be used for different refactorings in different parts of the code that might work with different types, we cannot expect the user to provide help for each individual scenario. The solution is using a dynamic instruction set that automatically adapts during the synthesis process based on context information. For the current example, we use the type information to restrict the instruction set such that the result computed by the refactored code is an integer.

While the introduction of dynamic instruction sets can be viewed as an engineering enhancement, we believe it can have a huge impact on the performance as well as the ease of use of program synthesizers.

#### (iii) Parametrize the search space

While the previous two directions were related to the size of the solution space, in this section, we discuss the actual search strategy and in particular a technique we found critical for the performance of the program synthesizer described in §4c. Remember that the target language in this work is the 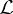-language given in figure 6.

Parametrizing the 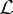-language induces a lattice of progressively more expressive languages. We start by attempting to synthesize a program at the lowest point on this lattice and increase the parameters of the language until we reach a point at which the synthesis succeeds. As well as giving us an automatic search procedure, this parametrization greatly increases the efficiency of our system because languages low down the lattice are very easy to decide safety for (i.e. the validation oracle finds an answer fast). If a program can be synthesized in a low-complexity language, the whole procedure finishes much faster than if synthesis had been attempted in a high-complexity language.

**Figure 10.**
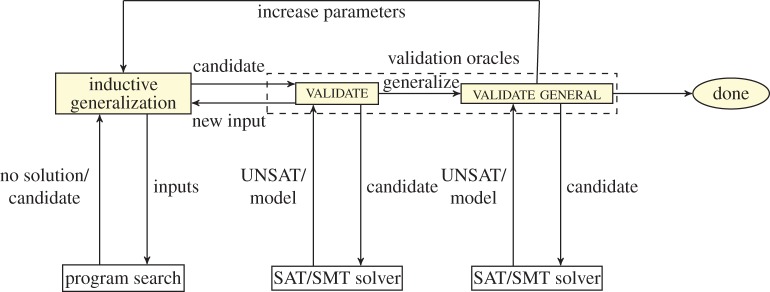
The CEGIS loop with solution generalization. (Online version in colour.)

We now adapt the CEGIS loop as illustrated in figure 10. We use the following parameters for our language 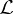:

— *Program length* (*l*). The first parameter we introduce is program length, denoted by *l*. At each iteration we synthesize programs of length exactly *l*. We start with *l* = 1 and increment *l* whenever we determine that no program of length *l* can satisfy the specification. When we do successfully synthesize a program, we are *guaranteed that it is of minimal length* because we have previously established that no shorter program is correct.
— *Word width* (*w*). An 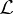-program runs on a virtual machine (the 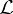-machine) that is parametrized by the *word width*, that is, the number of bits in each internal register and immediate constant.
— *Number of constants* (*c*). Instructions in 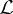 take up to three operands. As any instruction whose operands are all constants can always be eliminated (since its result is a constant), we know that a loop-free program of minimal length will not contain any instructions with two constant operands. Therefore, the number of constants that can appear in a minimal program of length *l* is at most *l*. By minimizing the number of constants appearing in a program, we are able to use a particularly efficient program encoding that speeds up the synthesis procedure substantially.

*Adjusting the search parameters*. The key to our automation approach is to come up with a sensible way in which to adjust the 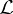-parameters in order to cover all possible programs. Two important components in this search are the adjustment of parameters and the generalization of candidate solutions. We discuss them both next.

**Figure 11.**
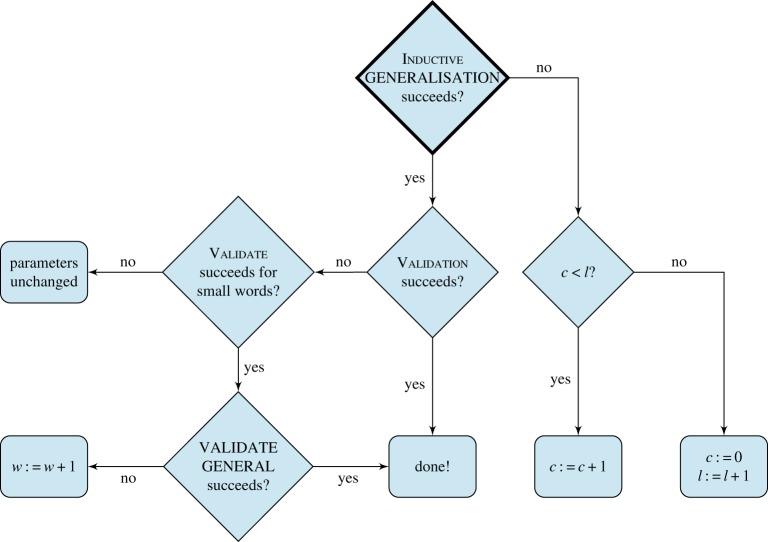
Decision tree for increasing parameters of 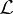 (taken from [9]). (Online version in colour.)

After each round of inductive generalization, we may need to adjust the parameters. The logic for these adjustments is given as a tree in figure 11. Whenever the inductive generalization fails, we consider which parameter might have caused the failure. There are two possibilities: either the program length *l* was too small, or the number of allowed constants *c* was. If *c* < *l*, we just increment *c* and try another round of synthesis, but allowing ourselves an extra program constant. If *c* = *l*, there is no point in increasing *c* any further. This is because no minimal 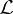-program has *c* > *l*, for if it did there would have to be at least one instruction with two constant operands. This instruction could be removed (at the expense of adding its result as a constant), contradicting the assumed minimality of the program. So if *c* = *l*, we set *c* to 0 and increment *l*, before attempting synthesis again.

If the inductive generalization succeeds but the first verification fails (i.e. validate in figure 10), we have a candidate program that is correct for some inputs but incorrect on at least one input. However, it may be the case that the candidate program is correct for all inputs when run on an 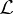-machine with a small word size (i.e. validate succeeds but validate general fails in figure 10). Thus, we try to generalize the solution to a bigger word size, as explained in the next paragraph. If the generalization is able to find a correct program, we are done. Otherwise, we need to increase the word width of the 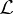-machine we are currently synthesizing for.

**Figure 12.**
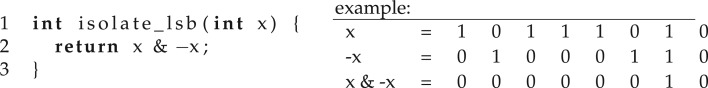
A tricky bitvector program (taken from [9]).

*Generalization of candidate solutions*. It is often the case that a program which satisfies the specification on an 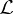-machine with *w* = *k* will continue to satisfy the specification when run on a machine with *w* > *k*. For example, the program in figure 12 isolates the least-significant bit of a word. This is true irrespective of the word size of the machine it is run on—it will isolate the least-significant bit of an 8-bit word just as well as it will a 32-bit word. An often successful strategy is to synthesize a program for an 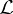-machine with a small word size and then to check whether the same program is correct when run on an 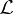-machine with a full-sized word.

**Figure 13.**
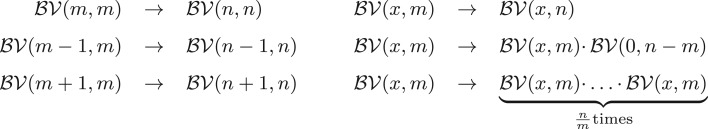
Rules for extending an *m*-bit wide number to an *n*-bit wide one (taken from [9]).

The only wrinkle here is that we will sometimes synthesize a program containing constants. If we have synthesized a program with *w* = *k*, the constants in the program will be *k*-bits wide. To extend the program to an *n*-bit machine (with *n* > *k*), we need some way of deriving *n*-bit wide numbers from *k*-bit ones. We have several strategies for this and just try each in turn. Our strategies are shown in figure 13. *BV*(*v*,*n*) denotes an *n*-bit wide bitvector holding the value *v* and *b·c* means the concatenation of bitvectors *b* and *c*. For example, the first rule says that if we have the 8-bit number with value 8, and we want to extend it to some 32-bit number, we would try the 32-bit number with value 32. These six rules are all heuristics that we have found to be fairly effective in practice.

#### (iv) Synthesis of multiple programs

**Figure 14.**
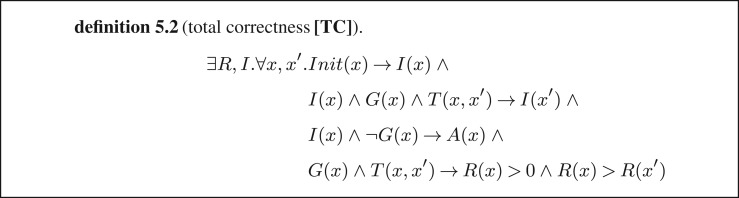
Formulae encoding total correctness of a loop.

There are often situations when the solution expected from the synthesizer is not represented by a single program, but by a set of such programs. For illustration, let us get back to using program synthesis for program analysis (see §4c) and, in particular, for proving total correctness of a program, i.e. proving that the program always terminates without any assertion violations. For this purpose, we consider the specification for the same loop *L*(*G, T*) as in §4c with initial states *Init* and assertion *A*. Similarly to §4c, we prove that *L* terminates by finding a ranking function, and that it is safe by finding a safety invariant. Correspondingly, we need to synthesize two programs, one computing the ranking function and one computing the safety invariant. The full specification is given in definition 5.2 (figure 14), where *R* is the ranking function and *I* is the safety invariant.

Given that synthesizing multiple programs makes the solution space grow exponentially, we have to come up with heuristics that facilitate concurrent synthesis of multiple programs. One such heuristic is described in [8] and consists of searching for multiple counter-examples in each verification phase of the CEGIS loop. For the current example, where we are interested in total correctness, this means that in each iteration of the CEGIS loop, the verification oracle will try to find two types of counter-examples: (i) a counter-example disproving that *I* is a safety invariant and (ii) a counter-example disproving that *R* is a ranking function. This ensures that the inductive generalization component receives sufficient information at each iteration to refine both synthesized programs, *I* and *R*.

The different types of counter-examples play an important role in the genetic programming backend (described in §3), which considers these counter-examples in its selection and crossover operators. Namely, candidate programs that satisfy the problem specification for inputs corresponding to distinct sets of counter-examples have a higher probability of selection as crossover partners in order to produce solutions that satisfy the specification for all possible inputs. This is preferable over fitness values based solely on the number of inputs for which the candidate is correct, since it avoids local minima where candidates may be correct for a multitude of inputs of one particular kind. In our experiments, we found that this choice of selection and crossover operators had considerably improved the performance of the inductive generalization phase.

#### (v) Improved counter-examples

The counter-examples provided by the verification oracle are used as the inputs of the inductive generalization engine. These counter-examples direct the inductive generalization process and, consequently, dictate how fast the CEGIS loop converges with a solution. The number of necessary counter-examples is related to the notion of teaching dimension from the learning theory. Informally, the teaching dimension of a concept class is the minimum number of instances a teacher must reveal to uniquely identify any target concept chosen from the class [37].

Our aim is to obtain a final solution in as few iterations as possible, i.e. we want to minimize the difference between the number of inputs required by the inductive generalization to find a solution and the teaching dimension. While Goldman & Kearns [37] showed that finding an optimal sequence of examples is equivalent to a minimum set cover problem, in this section, we discuss some heuristics for providing better counter-examples.

*Behavioural inductive synthesis.* Existent techniques using genetic programming as an inductive generalization strategy conventionally evaluate candidate programs using scalar performance measures that capture the number of passed or failed tests. While this is a natural objective measure of a program's performance, it does not make use of more detailed information on the program's behavioural characteristics. Thus, in order to drive the search process more efficiently, we suggest employing notions from behavioural program synthesis, which argues that programs are complex combinatorial structures that exhibit complex input–output behaviour that cannot be effectively reflected by a single scalar objective [38].

*Counter-example generalization.* Whenever the candidate solution found in the inductive generalization phase is not a solution for the whole problem (i.e. the validation oracle finds a counter-example), we need strategies for generalizing the counter-examples returned by the validation oracle in order to prune the search space as much as possible. One of the obvious candidate strategies for this is interpolation, which has been used in SAT-based model checking [39].

*Statistical fault localization.* Another technique that is worth investigating for its abilities to improve the inductive generalization strategy comes from statistical fault localization [40]. This has the potential to help direct the search by identifying the exact location in the synthesized program causing the specification to fail.

### (b) Solving process

CEGIS-based tools make heavy use of SAT/SMT solvers in the inductive generalization and validation phases in order to generate test inputs and find candidate solutions, respectively. Consequently, the solving process tends to be the most time-consuming part of the whole CEGIS process. In this section, we focus on ways of reducing the time taken by the solving process.

#### (i) Incremental SAT/SMT

One direction we are currently investigating is exploiting the incremental solving support offered by modern SAT/SMT solvers [41]. Incremental solving leverages the similarity across constraints to reduce overall solving cost. Rather than solving related formulae separately, modern solvers attempt to propagate information gathered during the solving process to future instances. Intuitively, solving a set of similar constraints in an incremental manner can be faster compared to solving each constraint in the set separately.

Recall from §3 that the formula whose satisfiability is checked by the inductive generalization phase is ∃***P***·∀***x*** ∈ inputs·*σ*(***P***,***x***). Then, if we assume this to be the formula checked in round *n* of the CEGIS loop, the formula in round *n* + 1 is

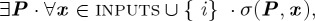

where *i* is the additional counter-example found in the validation phase of round *n*. These constraints can be rewritten as

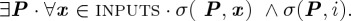


Note that the constraints generated in round *n* + 1 are identical with those generated in round *n* plus an extra conjunct. The search strategy that can directly employ incremental solving in order to take advantage of this similarity is the BMC backend. We believe that this has the potential to greatly improve performance.

#### (ii) Reduce context dependencies

While in the previous section we discussed a way of providing better solver support, in this section, we focus on simplifying the constraints that get sent to the solver.

For illustration, let us consider again using program synthesis in order to refactor code, and, in particular, we refer to our work in [6], where we refactored Java code handling collections. Note that the work in [6] follows the general architecture in figure 10.

Nearly every modern Java application constructs and processes collections. A key algorithmic pattern when using collections is iteration over the contents of the collection. We distinguish external from internal iteration. To enable external iteration, a Collection provides the means to enumerate its elements by implementing Iterable. Clients that use an external iterator must advance the traversal and request the next element explicitly from the iterator. The alternative to external iteration is internal iteration, where instead of controlling the iteration, the client passes an operation to perform to an internal iteration procedure, which applies that operation to the elements in the collection based on the algorithm it implements. In order to enable internal iteration, Java SE 8 introduces a new abstraction called Stream that lets users process data in a declarative way. The Stream package provides implementations of common internal iteration algorithms such as foreach, find and sort using optimized iteration orders and even concurrency where applicable. This enables users to leverage multicore architectures transparently without having to write multithread code.

We are interested in refactoring Java code handling collections to use Streams. For this purpose, the specification provided to the program synthesizer must check that the original and the refactored code are observationally equivalent. The challenge is that, while the actual code that gets refactored is generally not very large, the observational equivalence check may need to consider much larger units of code.

**Figure 15.**
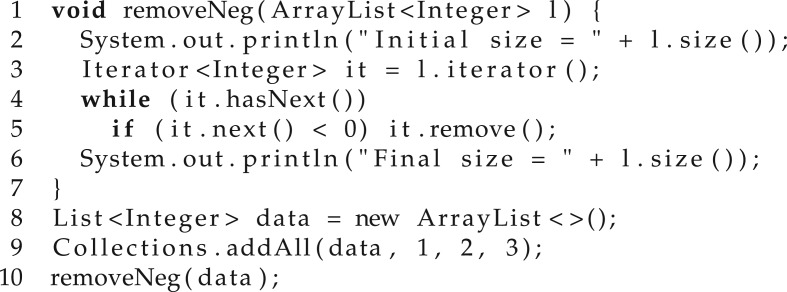
Filter example (taken from [6]).

For illustration, let us look at the example in figure 15. We define a method removeNeg that removes the negative values in the list received as argument, which we later call for the list data. Now, let us assume that we want to refactor removeNeg, which currently uses external iteration to use streams. The question is which lines of code do we consider for the equivalence check? Is it just lines 3–5 that actually iterate over the collection, the whole removeNeg method or the whole code in figure 15? Note that if we choose the last option, given that data contains only positive values, applying removeNeg does not have any effect. Thus, for this particular calling context, we could refactor the body of removeNeg to a NO-OP. While this refactoring is correct for the code given in figure 15, it may not be so for different calling contexts.

In order to simplify the equivalence check and consequently, the constraints to be solved by the validation oracle, we decided to synthesize general refactorings that are correct independent of their context. This enables the equivalence checking procedure to only consider the actual code being refactored (lines 3–5 in the current example) and completely ignore the rest.

For this purpose, we over-approximate the context of the code to be refactored by nondeterministically picking the initial configuration (i.e. we nondeterministically assign all the variables and the contents of collections). For the current example, this means that we nondeterministically pick the contents of the list l. This over-approximates the context in the sense that it may require us to consider more initial configurations than those reachable at the start of the actual user code to be refactored. Consequently, when checking equivalence, we only need to consider the variables (and collections) that are accessed by the exact code to the refactored (as opposed to all the live program variables).

While our decision to only consider general refactorings drastically reduced the time taken by the solving process, there were certain aspects that we had to consider, with the main one being aliasing: are any side-effects due to aliasing that we are not considering? The answer is no, our approach is safe for pointer variables. The reason is that the only two potential aliasing scenarios involving a pointer variable *p* that is not directly used by the code to be refactored Code, are the following:

(1) *p* points to a collection that is modified by Code. As the refactoring is going to perform an equivalent transformation in-place, the refactoring will be transparent to *p*.
(2) *p* is an iterator over a collection accessed by Code. Then, if Code modifies the collection, so will the refactored code, which will result in *p* being invalidated in both scenarios. Contrary, if Code does not modify the collection, neither will the refactored code, and *p* will not be affected in either one of the cases.

Next, we illustrate scenario 1 by considering again method removeNeg in figure 15 with the following calling context, where we assume *p* points to some list and we create an alias *P*′ of *P*:


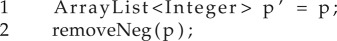


At a first glance, a potential refactoring for lines 3–5 in removeNeg is:


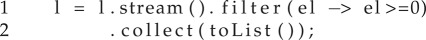


However, this is incorrect when using the refactored function in the calling context mentioned above: While the list *P* points to is correct, the list pointed by *P′* is not updated. Thus, after the call to removeNeg, *p* will correctly point to the filtered list, whereas *P′* will continue pointing to the old unfiltered list. To avoid such situations, we perform refactorings of code that mutates collections in-place. Thus, a correct refactoring for method removeNeg is:


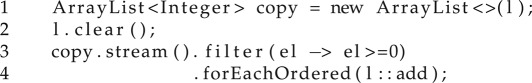


Here, we first create a copy of l. After performing the filtering on copy, we use forEachOrdered, provided by the Stream API, to add each element of the temporary stream back to the list pointed to by l (in the order encountered in the stream). Thus, we are not creating a new list with a new reference, but using the original one, which makes the refactoring transparent to the rest of the program, regardless of potential aliases.

## 6. Conclusion

In this paper, we discussed one of the recent directions in program synthesis, namely counter-example guided inductive synthesis (CEGIS). While program synthesis has the advantage of generality by providing a unified solution to a very large class of problems, the downside is that it may be challenging for program synthesis-based tools to match the performance of their special-purpose counterparts. After looking at ways in which current CEGIS-based approaches tackle this performance issue, we discussed some other potential directions to follow up on. These directions are based on our experience with CEGIS and our conjecture is that further investigating them will help reduce the performance penalty specific to program synthesis in a manner that is transparent to the user and does not restrict the class of program synthesis applications.

## Footnotes

1 ***P*** stands for a vector, where we refer to each individual element as *P*.
